# YOD1 serves as a potential prognostic biomarker for pancreatic cancer

**DOI:** 10.1186/s12935-022-02616-9

**Published:** 2022-05-31

**Authors:** Zhishuo Zhang, Wenxia Zhao, Yiming Li, Yang Li, Hanzeng Cheng, Liyun Zheng, Xiaoyu Sun, Hao Liu, Rongguang Shao

**Affiliations:** 1 Department of Organ Transplantation and Hepatobiliary Surgery, The First Hospital of China Medical University, Shenyang, Liaoning People’s Republic of China; 2 NHC Key Laboratory of Antibiotic Bioengineering, Laboratory of Oncology, Institute of Medicinal Biotechnology, Peking Union Medical College and Chinese Academy of Medical Sciences, 1 Tiantan Xili, Beijing, 100050 China; 3 Beijing Key Laboratory of Active Substance Discovery and Druggability Evaluation, Institute of Materia Medica, Peking Union Medical College and Chinese Academy of Medical Sciences, Beijing, 100050 China; 4 School of Pharmacy, Department of Pharmacology, China Medical University, Liaoning Key Laboratory of Molecular Targeted Anti-Tumor Drug Development and Evaluation, Shenyang, Liaoning China

**Keywords:** YOD1, OTUD2, Pancreatic cancer, Prognosis, Enrichment analysis, Biomarker

## Abstract

**Background:**

Ubiquitination is a basic post-translational modification of intracellular proteins and can be reversed enzymatically by DUBs (deubiquitinating enzymes). More than 90 DUBs have been identified. Among them, the deubiquitinating enzyme YOD1, a member of the ovarian tumor domain protease (OTUs) subfamily, is involved in the regulation of endoplasmic reticulum (ER)-related degradation pathways. In fact, it is reported that YOD1 is an important proliferation and metastasis-inducing gene, which can stimulate the characteristics of cancer stem cells and maintain circulating tumor cells (CTC). However, the expression level, prognostic effect and biological functional mechanism of YOD1 in pancreatic cancer are still unclear.

**Results:**

In the GEO and TCGA databases, YOD1 mRNA expression is significantly up regulated in a variety of human pancreatic cancer tissues. Survival analysis showed that the up regulation of YOD1 can predict poor prognosis of pancreatic cancer. Cox analysis showed that high YOD1 expression is an independent prognostic factor of pancreatic cancer. ROC analysis shows that YOD1 has significant diagnostic value. The immunohistochemistry (IHC) results showed that the protein expression level of YOD1 in pancreatic cancer tissue was higher than that in neighboring non-pancreatic cancer tissues (*P* < 0.001). In addition, we found that YOD1 expression is negatively correlated with the infiltration level of CD8 + T cells, macrophages, neutrophils and dendritic cells (DC) in pancreatic cancer. The expression of YOD1 has a strong correlation with the different immune marker sets in PAAD. Co-expression network and functional enrichment analysis indicate that YOD1 may participate in the development of pancreatic cancer through cell adhesion molecules, p53, Hippo, TGF-β and other pathways. The experimental results of EDU, Transwell, Immunohistochemistry (IHC), Western blot and Flow Cytometry indicate that YOD1 is highly expressed in pancreatic cancer cells and pancreatic cancer tissues, and its overexpression can promote the proliferation and metastasis of pancreatic cancer cells and affect the immune microenvironment.

**Conclusion:**

Our results indicate that YOD1 may be a useful biomarker for the prognosis of human pancreatic cancer, and it may also be a potential molecular target for the diagnosis and treatment of pancreatic cancer.

**Supplementary Information:**

The online version contains supplementary material available at 10.1186/s12935-022-02616-9.

## Background

Pancreatic adenocarcinoma (PAAD) is a kind of malignant gastrointestinal system carcinoma characterized with difficult early diagnosis, complex treatment and highly poor prognosis. 80–90% of these tumors are Pancreatic Ductal Adenocarcinoma (PDAC). According to the American Cancer Society, there were 57,600 new cases and 47,050 new deaths from PAAD in 2020. The five-year survival rate for PAAD is about 9%, the lowest of all cancers [1]. At present, surgery is the main radical treatment for early PAAD, while chemotherapy is the main comprehensive treatment for locally advanced or distant metastatic PAAD [2]. However, during chemotherapy, many PAAD patients show resistance to multiple antitumor therapies, leading to rapid progression of the disease and low rate of complete response [3]. Although great progress has been made in the molecular characterization of PAAD, the regulatory mechanisms of many important genes are still unclear. Early diagnosis and early resection are important to improve the survival rate of PAAD patients, therefore, it is of great significance to find and study the early diagnosis and prognostic markers of PAAD for improving the survival rate of PAAD patients and searching for new drug targets.

Protein ubiquitination is the post-translational modification of most Lys residues and regulates many cellular processes, including protein degradation, intracellular transport, cell signaling, autophagy, transcription, translation and DNA damage responses [4]. These diverse functions are achieved through the ability of ubiquitin (Ub) which can form topologically different signals. The deubiquitination enzyme (DUB) can remove Ub modification and regulate almost all Ub-dependent processes [5]. In total, about 90 DUBs are encoded in the human genome and are closely associated with many human diseases, such as neurodegeneration, inflammation, infection and cancer.

Ovarian tumor-associated protease (OTU), a subtype of DUB, is a modulator of important signaling cascades with strong ubiquitin chain recognition specificity. According to sequence similarity, OTU can be divided into four subfamilies: the OTUB subfamily/Otubains, the OTUD subfamily, the A20-like subfamily and the OTULIN subfamily. The OTUD gene family consists of 8 members: OTUD1, OTUD2/YOD1, OTUD3, OTUD4, OTUD5, OTUD6A, OTUD6B and ALG13 [6].

The OTUD gene family plays an important role in tumor genesis, metastasis and invasion. OTUD1 inhibits breast cancer metastasis by reducing TGF-β-induced tumor-promoting responses by deubiquitinating SMAD7 [7]. OTUD1 is negatively regulated by miR-4429 in ovarian cancer, and the expression of YOD1 increases when miR-4429 is reduced, promoting the growth and metastasis of ovarian cancer. OTUD3 can promote the occurrence of lung cancer by stabilizing GRP78 (The glucose-regulated protein 78 kDa, Bip) [8], OTUD4 [9], OTUD5 [10], and OTUD6B [11] also play important roles in the pathogenesis of different tumors. However, there are few studies related to the OTUD family in PAAD, little is known about the correlation between the expression of OTUD family members and the clinical and pathological parameters of PAAD, and the prognostic potential of OTUD as a biomarker in PAAD remains unknown. Therefore, the differential expression of OTUD and their prognostic value in PAAD patients need to be further studied. In this study, this essay explored a large sample-based database to fully analyze the role of the OTUD family in pancreatic cancer.

## Materials and methods

### Public database mining of the OTUD gene family

#### ONCOMINE database

ONCOMINE database (www.oncomine.org) is an integrated online cancer microarray database for DNA sequence analysis. Gene expression analysis and DNA microarrays are powerful methods for studying cancer transcripts [12]. In our study, transcriptional levels of 8 different OTUD family members in different cancer tissues and their corresponding normal control samples were obtained from the ONCOMINE database. Differences in transcriptional expression were determined by the T test. The standard ranges are *P*-value < 0.05, multiple change: < 2.0.

#### TCGA and GTEx, GEO, GEPIA, MERAV databases verified the expression differences

We used microarray series GEO datasets to compare the expression of OTUD1, YOD1, OTUD3, OTUD4, OTUD5, OTUD6B in single series datasets through SangerBox online website (http://sangerbox.com/), so as to comprehensively investigate the expression of OTUD gene family in PAAD, *P* < 0.05, FC > 2 was considered statistically significant.

The expression data of normal people were downloaded from GTEx, and the expression data of PAAD patients were downloaded from TCGA. Perl software and the R software "limma" R package were used for processing and merging to determine whether there were differences in the expression of OTUD family between PAAD and normal pancreatic tissue. *P* < 0.05, |Log2FC|> 1 was considered statistically significant.

Then we conducted a secondary analysis and verification from GEPIA and MERAV databases, the website GEPIA (http://gepia.cancer-pku.cn/) can be used to comprehensively compare and analyze the expression distribution of OTUD gene family in the TCGA and GTEx databases in PAAD tissues and normal tissues [13]. *P* < 0.05, |Log2FC|> 1 was the criteria for screening differential genes in GEPIA. Metabolic Generapid Visualizer (MERAV) was used to analyze the expression of OTUD gene family in primary PAAD tissues, PAAD cell lines and adjacent normal tissues [14].

#### cBioPortal

cBioPortal (www.cbioportal.org) is a publicly available website for analyzing and visualizing cancer genomics data [15]. In this study, we analyzed the mutation of gene markers in PAAD of 8 members of the OTUD gene family which include Mutations, Putative copy-number alterations from GISTIC and mRNA expression z-scores relative to diploid samples (RNA Seq V2 RSEM).

#### Human Protein Atlas

Human Protein Atlas (HPA, https://www.proteinatlas.org) use transcriptomics and proteomics techniques to identify tumor-type specific Protein expression patterns that are differentially expressed in specific types of tumors [16]. In this study, differences in YOD1 protein expression between PAAD tissue and its corresponding normal tissue were directly compared using immunohistochemical images.

## Survival analysis and independent prognostic factors analysis

Using the Kaplan–Meier plotter (http://kmplot.com/analysis/) [17] and according to the expression of YOD1, patients were divided into high-risk groups and low-risk groups, the risk curves of Overall Survival (OS) and Disease Free Survival (DFS) were drawn. Later, the second verification is carried out based on the data of TCGA and GTEX.

Transcriptional and clinical data of PAAD patients were downloaded from the TCGA website, and then we classified patients into high-risk groups and low-risk groups according to the median. Univariate and multivariate Cox regression analysis was performed using the “survival” R package to determine whether the gene can be used as an independent prognostic factor, and *P* < 0.05 was considered statistically significant.

## Stemness index correlation, immune cell infiltration and tumor microenvironment analysis

### Immune cell infiltration analysis

Data were downloaded from TCGA and according to the different expression levels of YOD1 in PAAD, the median was cut off and divided into two groups of high and low risk. The single-sample gene set enrichment analysis (ssGSEA) method was used to score the high and low risk groups of the specified genes respectively, and the immune cell infiltration in the high and low risk groups were analyzed by R language, and *P* < 0.05 was considered significant.

Vesteinn Thorsson et al. determined 6 immune subtypes by performing immunogenome analysis on 10,000 samples, namely: C1: wound healing, C2: IFN-γ dominant, C3: inflammatory, and C4: lymphocyte depleted, C5: immunologically quiet, C6: and TGF-β dominant [18]. “limma” R package was used to analyze the correlation between high expression of YOD1 and 6 immune subtypes.

### Correlation analysis of tumor microenvironment

In order to predict whether the increase of YOD1 expression in PAAD patients would affect the tumor microenvironment, we applied the ESTIMATE algorithm to calculate the immune cell score and stromal cell score of each PADD patient in the TCGA database [19]. R value and *P* value were calculated according to the ESTIMATE score and data of high and low risk groups. *P* value < 0.05 was considered significant, R value > 0 is positive correlation and R < 0 is negative correlation.

### Correlation analysis of stemness index

Malta et al. used one-class logistic regression (OCLR) machine-learning algorithm to introduce stemness index to assess the stem cell characteristics of each tumor sample in the Cancer Genome Atlas (TCGA) database, we found that these indices could accurately predict metastatic events and explain intra-tumor heterogeneity [20]. Therefore, we extracted mRNA expression data of tumor samples from TCGA database to calculate stem cell index (mRNAsi) of different risk groups. Then spearman algorithm was used for statistical analysis to detect the correlation between cancer stem cell characteristics and YOD1 expression, and R values and *P* values were calculated. *P* < 0.05 is significant, and R value > 0 is positive correlation, R value < 0 is negative correlation, and visualization.

## Biological function analysis of YOD1

### Gene set enrichment analysis (GSEA)

Based on the Gene Ontology (GO) database and Kyoto Encyclopedia of Genes and Genomes (KEGG), the GSEA method was used for the enrichment analysis of the high expression and low expression groups of specific genes. In this study, the gene sets of GSEA v.4.0 c2.all.v7.4.symbols.gmt [Curated] and c2.cp.kegg. v7.4.symbols.gmt [Curated] were analyzed. *P*-value < 0.05 and the enrichment results of false detection rate (FDR) < 0.25 were considered statistically significant. And we ranked consequences from high to low, according to NSE.

### LinkedOmics analysis

The Linkedomics database (http://www.LinkedOmics.org/login.php) is a web-based platform for analyzing 32 cancer datasets from TCGA [21]. The LinkFinder module of Linkedomics was used to study YOD1-related differentially expressed genes in the TCGA PAAD cohort (n = 307). Pearson correlation coefficient was used for statistical analysis of the genes co-expressed with YOD1 in PAAD, and the results were shown in the heat map. In order to further study the biological function of YOD1, we use TCGA data to perform single-gene COX analysis on genes that are positively related to YOD1 And use the "clusterProfiler" R package for GO, KEGG enrichment analysis, and the result is drawn as bubble charts.

## Confirmatory experiment

### Patients and specimens

A total of 6 normal tissues, 6 cancer tissues which from the same cohort of cancer patients were collected from 6 cancer patients who come from The first hospital of China Medical University between 2020 and 2021. After informed consent had been obtained, the histopathological diagnosis, stage, and grade of PAAD were based on the FIGO classification. The clinical data of PAAD patients are shown in Additional file 2: Table S1. This study was approved by the Ethics Committee of the First Hospital of China Medical University.

### Materials

Both pCMV6-YOD1 gene expression plasmid and its control plasmid were purchased from Beijing sinobiological. siRNA: siRNA targeting YOD1 gene and control siRNA were synthesized by Guangzhou ribobio. Edu (5-ethynyl-2′-deoxyuridine) detection kit (#C10310-3) was purchased from Guangzhou ribobio. Transwell Chamber was purchased from Corning, Inc., USA. Crystalline purple dye solution, BCA protein detection kit (enhanced version) and Cell cycle detection kit (C1052) were purchased from Beyotime Biotechnology. In immunohistochemistry part, HRP-conjugated second antibody (#5020) and DAB (#00031) was purchased from MXB company (Fuzhou, China). Anti-CD3 antibody (GB13314) and Anti-CD8 antibody (GB13429) was purchased from Servicebio company (Wuhan, China). Anti-YOD1 antibody (#25370-1-AP) was purchased from Proteintech company (Wuhan, China).

### Detection of cell proliferation in 5-Ethynyl-2'-deoxyuridine (EdU) assay

Cells were inoculated on 24-well glass slides at a density of 50,000 cells/500 μL and cultured until normal growth stage. After the cells were fixed, add 300 μL PBS solution (osmotic agent) containing 0.5% Triton X-100 to each well, and incubate in a decolorizing shaker for 10 min. They were then stained with Apollo. Immediately, the result was observed, photographed and counted with Olympus fluorescence inverted microscope, and the experiment was repeated for three times.

### Western blot

The cells were lysed with RIPA cell lysate (containing 1% PMSF). Subsequently, BCA method was used to determine the protein concentration. Proteins were isolated using SDS–polyacrylamide gel electrophoresis (SDS-PAGE) and transferred to PVDF membrane (Millipore, Burlington, US). Then use 5% skim milk powder to block. After the blocking is completed, the membrane was immunoblotted with the specified antibody, and the enhanced HRP substrate chemiluminescence solution (ECL) was used for color imaging in a gel imager.

### Transwell

After the chambers were balanced with serum-free medium for 30 min, the AsPC-1 and MIA-PaCa-2 cells in logarithmic growth phase after transient transfection for 24 h were digested. The upper compartment was inoculated with 30,000/200 μL serum-free medium and the lower compartment was inoculated with 20%FBS. According to the needs of the experiment, 4% paraformaldehyde was used for fixation at 24 h and 48 h respectively, and crystal violet staining was used. For a 6 h short-term observation, the upper compartment was inoculated with 300,000/200 μL serum-free medium. After the completion, the chamber was observed and photographed with Olympus inverted fluorescence microscope. The chamber was cut off with a blade, dissolved in 33% acetic acid, and the absorbance value at 570 nm was measured with a microplate analyzer. The experiment was repeated for three times.

### Flow cytometry

AsPC-1 and MIA-PaCa-2 cells in logarithmic growth phase after transient transfection for 48 h were digested. Add 1 mL of pre-chilled PBS, resuspend the cells. Add 1 mL of pre-chilled 70% ethanol and fix overnight. The next day, the cells were resuspended with 1 mL of pre-chilled PBS and the freshly prepared propidium iodide staining solution was added to fully resuspend the cells. After incubation at 37 °C for 30 min in the dark, detection was performed at 488 nm with FACS Calibur, BD.

### Immunohistochemistry (IHC)

Paraffin section of six patients were first deparaffinized and heated in pressure cooker for 10 min. After cooling to room temperature, the sections were immersed for 5 min in PBS three times and followed by endogenous peroxidase activity blocking with 3% H_2_O_2_ and non-specific staining blocking with 10% goat serum. Then these sections were incubated with primary antibodies (YOD1 1:300 diluted, CD3 1:200 diluted, CD8 1:500 diluted) overnight at 4 ℃ and treated for 30 min at 37 ℃ with second antibody. The sections were staining for 2 min by using DAB and rinsed off in deionized water to terminate DAB reaction. Then using traditional method to evaluate under the optical microscope.

### Statistical analysis

In this study, R software v 4.0.5 was used for statistical analysis. The receiver operating characteristic (ROC) curve is used to analyze and evaluate the diagnostic value. The Kaplan–Meier curve and log-rank test were used for prognostic analysis. The univariate and multivariate Cox proportional hazard models were used to calculate HR and 95% CI. *P* < 0.05 was considered statistically significant. ImageJ software’s IHC Profiler plugin was used to automate the scoring of sample staining for IHC.

## Results

### In the OTUD gene family, only YOD1 was stably and highly expressed in PAAD

**Fig. 1:**
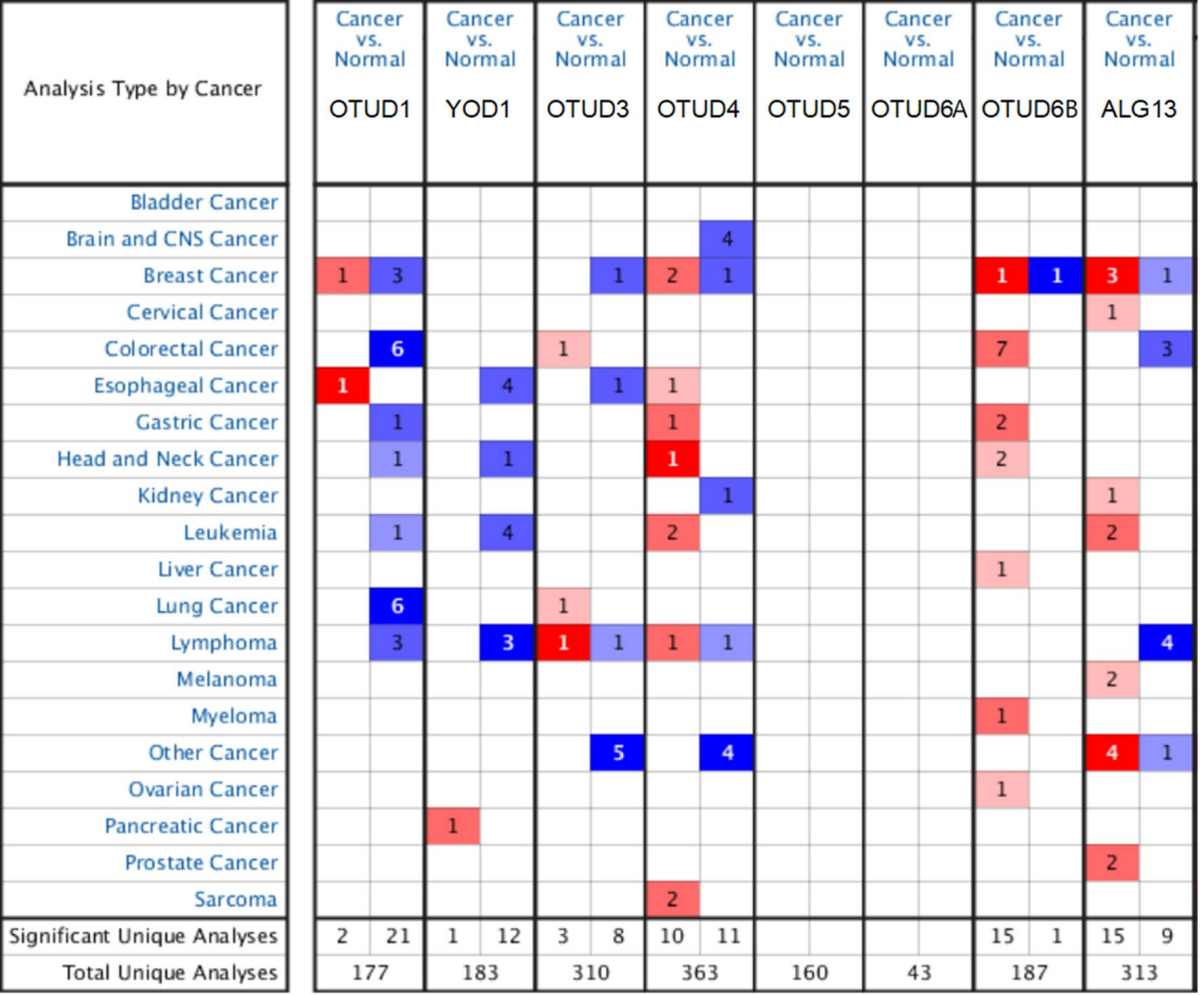
The transcription levels of OTUD family members in different types of cancers (ONCOMINE). The graph shows the numbers of datasets with statistically significant mRNA over-expression (red) or down-regulated expression (blue) of the target gene. The threshold was designed with following parameters: *p*-value 0.05 and fold change of 2.0

In order to explore the prognosis and potential clinical value of different OTUD gene families in PAAD patients, we firstly analyzed the expression of OTUD gene families in PAAD which using ONCOMINE. As shown in Fig. 1 and Table 1, the mRNA expression of YOD1 in PAAD was significantly higher than that in normal tissues (*P* = 4.43E−8). Other members of the OTUD gene family had no significant difference. Then we used R software to analyze TCGA combined with GTEx data, and the results were shown in Table 2. In PAAD, the expressions of OTUD1 (*P* = 1.06E−52), YOD1 (*P* = 2.20E−50) and OTUD5 (*P* = 1.53E−45) in tumor tissues were higher than those in normal tissues. In addition, we also conducted dual validation in tumor tissues and cell lines through MERAV and GEPIA websites. In MERAV, the expressions of YOD1, OTUD4 and OTUD6b in tumor tissues and cell lines were different from those in normal tissues (Additional file 1: Fig. S1). In GEPIA, the expressions of OTUD1, YOD1, and OTUD5 were higher in tumor tissues than in normal tissues (*P* < 0.05) (Additional file 1: Fig. S2). Compared with different databases, only YOD1 in the OTUD gene family was found to be stably and highly expressed in PAAD.

**Table 1:** Differences in the transcription level of the OTUD family in pancreatic cancer and normal pancreatic tissues (ONCOMINE: gene rank < 10%)

Gene	Pathology	Fold change	P-value	*t*-Test	References
OTUD1	PDAC	1.786 –1.347	8.90E-4 0.029	3.313 –1.938	Ishikawa pancreas [24] Ishikawa pancreas [24]
YOD1*	PC	2.386	4.43E-8	6.695	Pei pancreas [36]
OTUD3	PDAC	1.438	0.041	1.836	Grutzmann pancreas [11]
OTUD4	\	\	\	\	\
OTUD5	PDAC	1.415	2.29E-4	4.783	Buchholz pancreas [8]
OTUD6A	\	\	\	\	\
OTUD6B	PAAD	1.079	6.18E-5	4.386	TCGA pancreas [32]
ALG13	PDAC	− 1.911	0.031	− 1.911	Ishikawa pancreas [24]

*PDAC* pancreatic ductal adenocarcinoma, *PC* pancreatic cancer, *PAAD* pancreatic adenocarcinoma

**Table 2:** Differences in the transcription level of the OTUD family in pancreatic cancer and normal pancreatic tissues (TCGA and GTEx)

Gene	ConMean	TreatMean	Log2FC	*P*-Value	FDR
OTUD1*	2.34	4.82	2.47	1.06E−52	6.36E−52
YOD1*	1.20	2.56	1.35	2.20E−50	8.75E−50
OTUD3	1.95	1.61	− 0.33	7.10E−14	9.13E−14
OTUD4	\	\	\	\	\
OTUD5*	9.94	13.71	3.76	1.53E−45	4.15E−45
OTUD6A	\	\	\	\	\
OTUD6B	1.77	2.19	0.41	1.17E−08	1.39E−08
ALG13	7.57	2.89	− 4.68	4.72E−53	3.16E−52

**Fig. 2:**
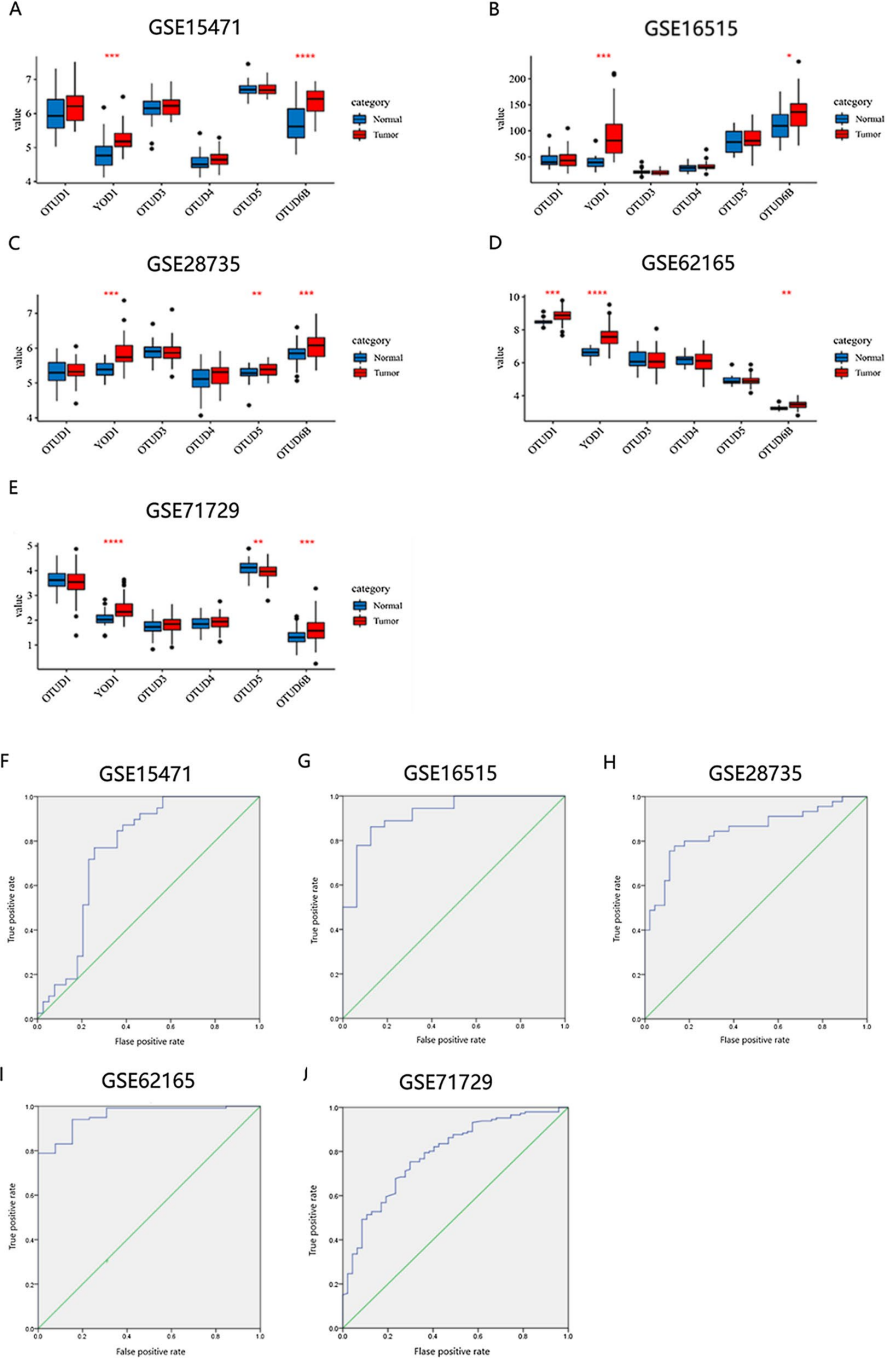
The GEO database compares the transcription levels of OTUD family members in PAAD and analyzes the diagnostic potential of YOD1. **A–E** GSE15471, GSE16515, GSE28735, GSE62165, GSE71729. **F**–**K** ROC curves corresponding to (**A**–**E**)

To further verify the expression of YOD1 gene, we downloaded and analyzed five data sets from the GEO database: GSE15471, GSE16515, GSE28735, GSE71729, and GSE62165 included 278 tumor samples and 186 normal tissues. Results showed that the expression level of YOD1 was significantly increased in PAAD tissues compared with normal tissues. The receiver operating characteristic curve (ROC curve) was used to verify the diagnostic effectiveness, and the results showed that the AUC was in the range of 0.61–0.985 (*P* = 0.001), with good diagnostic effectiveness (Fig. 2).

**Fig. 3:**
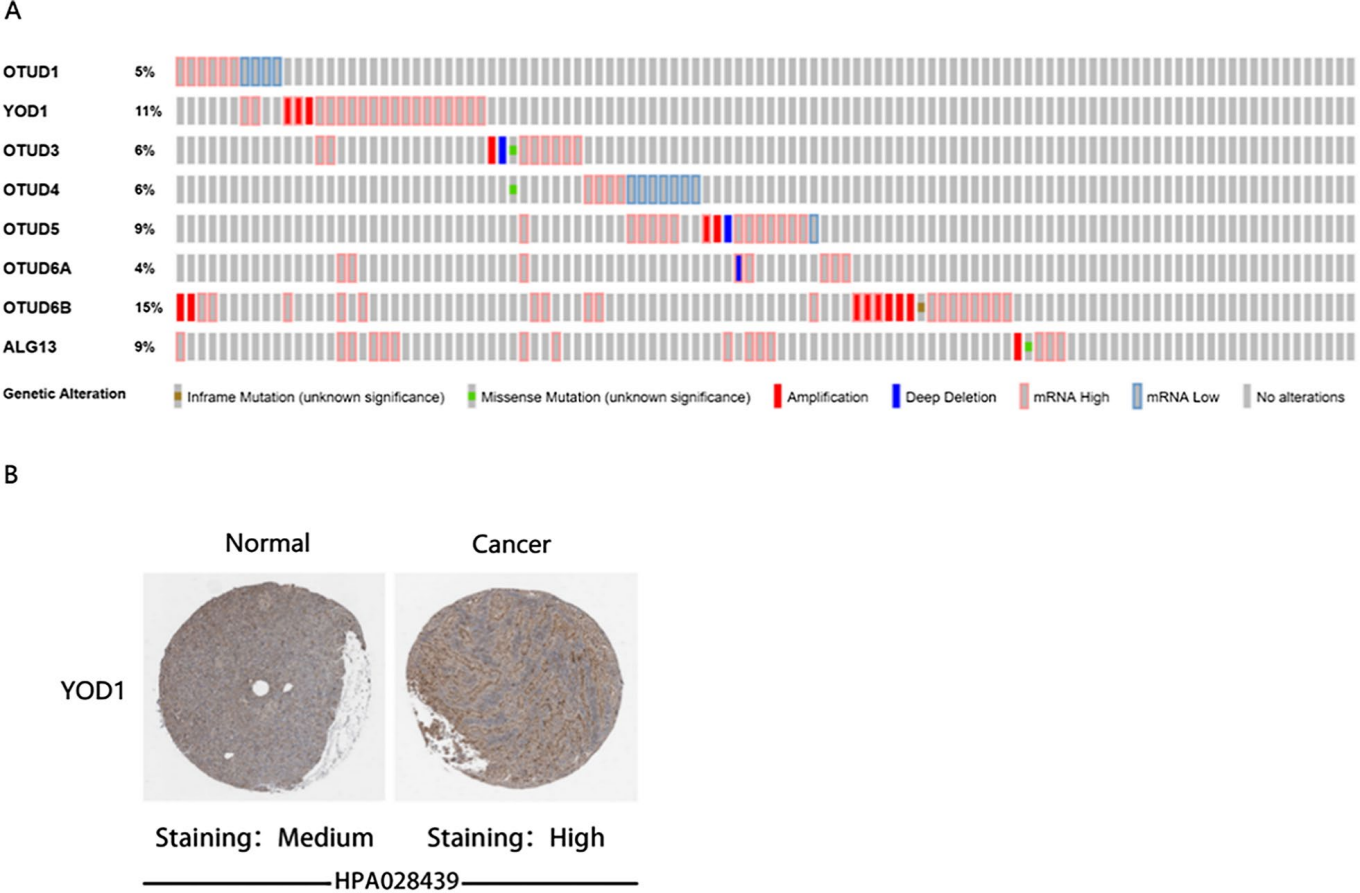
The mRNA level and the cancer tissue protein level of pancreatic cancer patients are higher in YOD1. **A** Genetic Alteration frequency of OTUD family members in PAAD (cBioPortal). **B** Protein expression patterns of YOD1 in normal tissue and pancreatic cancer tissue (Human Protein Atls)

In addition, we used the cBioPortal website analysis and found that in PAAD, the percentage of changes of genetic markers in the OTUD gene family varied from 5 to 15% (OTUD1, 5%; YOD1, 11%; OTUD3, 6%; OTUD4, 6%; OTUD5, 9%; OTUD6A, 4%; OTUD6B, 15%; ALG13, 9%), and it is worth noting that the high proportion of YOD1 mRNA was observed (Fig. 3A).

HPA uses proteomic techniques to identify tumor-type specific protein expression patterns that are differentially expressed in specific types of tumors. After the analysis found that only YOD1 mRNA level was stable and high in PAAD, we tried to explore the protein expression pattern of YOD1 in PAAD by HPA. Immunohistochemical results showed that YOD1 was highly expressed in PAAD compared to normal pancreatic tissue at the protein level (Fig. 3B).

In summary, our results indicate that the transcription and protein expression of YOD1 are stably and highly expressed in PAAD patients.

### The high expression of YOD1 is an independent factor affecting the poor prognosis of PAAD

**Fig. 4:**
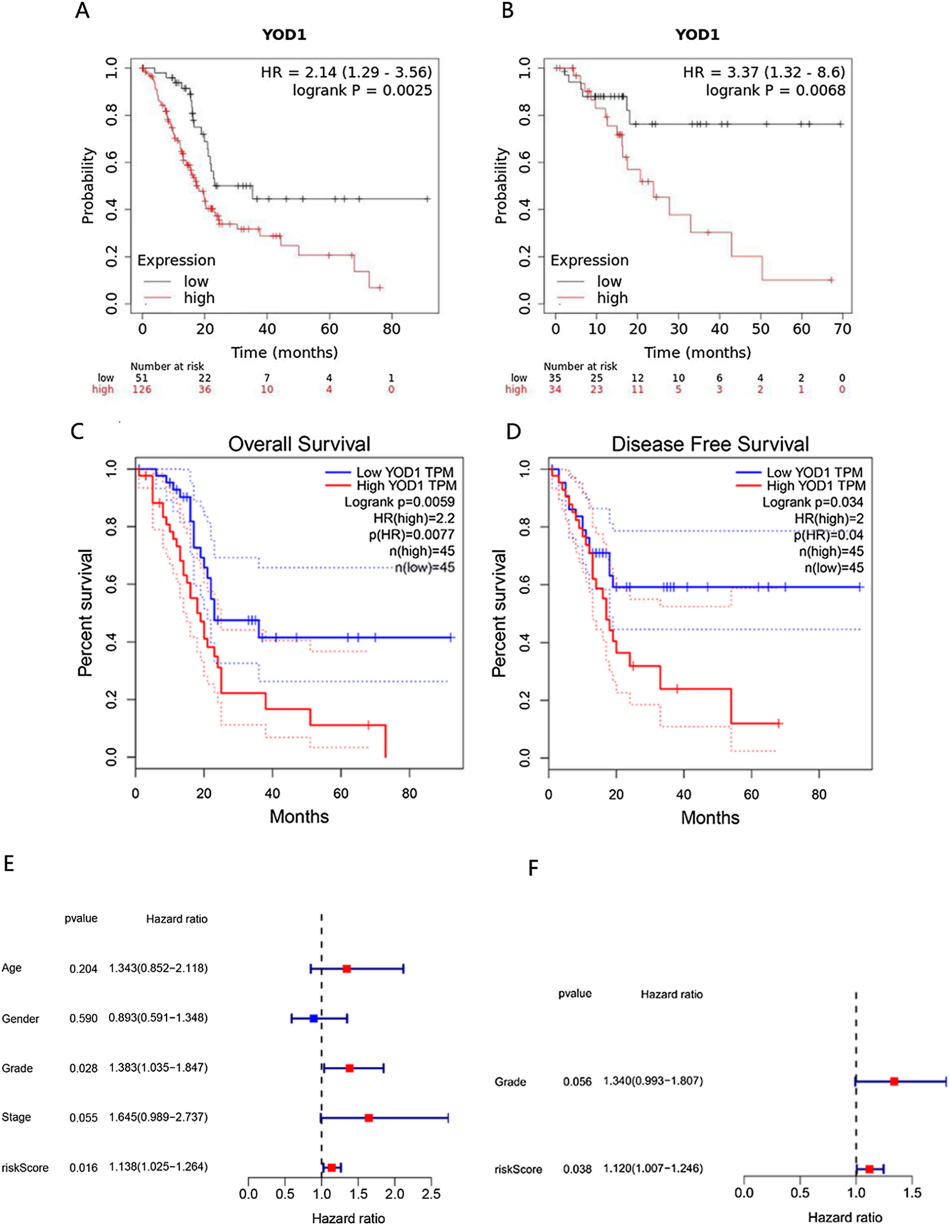
The prognostic value of mRNA level of YOD1 (Kaplan–Meier plotter) and Univariate and multivariate Cox regression analysis in PAAD patients. **A**, **B** The OS and DFS survival curves comparing patients with high (red) and low (black) YOD1 expression in PAAD were plotted using Kaplan–Meier plotter database at the threshold of p-value of < 0.05. **C**, **D** The OS and DFS survival curves comparing patients with high (red) and low (blue) YOD1 expression in PAAD were plotted using GEPIA database at the threshold of p-value of < 0.05. **E** Forest plot of Univariate COX regression analysis in PAAD from TCGA database. **F** Forest plot of multivariate COX regression analysis in PAAD from TCGA database

We used Kaplan–Meier plotter to analyze 177 PAAD samples and found that for overall survival (OS), YOD1 hazard ratio (HR) = 2.14 (1.29–3.56, *P* = 0.0025) and for relapse-free survival (RFS), YOD1 HR = 3.37 (1.32–8.6, *P* = 0.0068) (Fig. 4A, B). Therefore, the high expression of YOD1 is significantly positively correlated with the poor prognosis of PAAD patients. We also obtained the same results in GEPIA database. Compared with the low-expression group, the OS (HR = 2.2, *P* = 0.0077) and DFS (HR = 2, *P* = 0.04) of the group with high expression of YOD1 decreased (Fig. 4C, D).

In addition, univariate and multivariate Cox regression analyses were performed on PAAD patient data from the TCGA database. The hazard ratio (HR) of the risk score and 95% CI were 1.138 and 1.025–1.264 (*P* = 0.016), and 1.120 and 1.007–1.246 (*P* = 0.038), this indicates that the YOD1 risk model can be used as an independent prognostic factor. (Fig. 4E, F) The higher the expression of YOD1 is, the worse the prognosis of PAAD patients are.

These results suggest that YOD1 mRNA expression is significantly associated with prognosis in patients with PAAD, and they may be used as a potential biomarker to predict survival in PAAD patients.

### Correlation analysis of YOD1 tumor immune microenvironment and stemness index

The PAAD microenvironment has two main characteristics: dense connective tissue hyperplasia and extensive immunosuppression. Due to the specific characteristics of the immune microenvironment, tumor tissue is prone to survival and insensitive to chemoradiotherapy, so it results the poor prognosis [22].

**Fig. 5:**
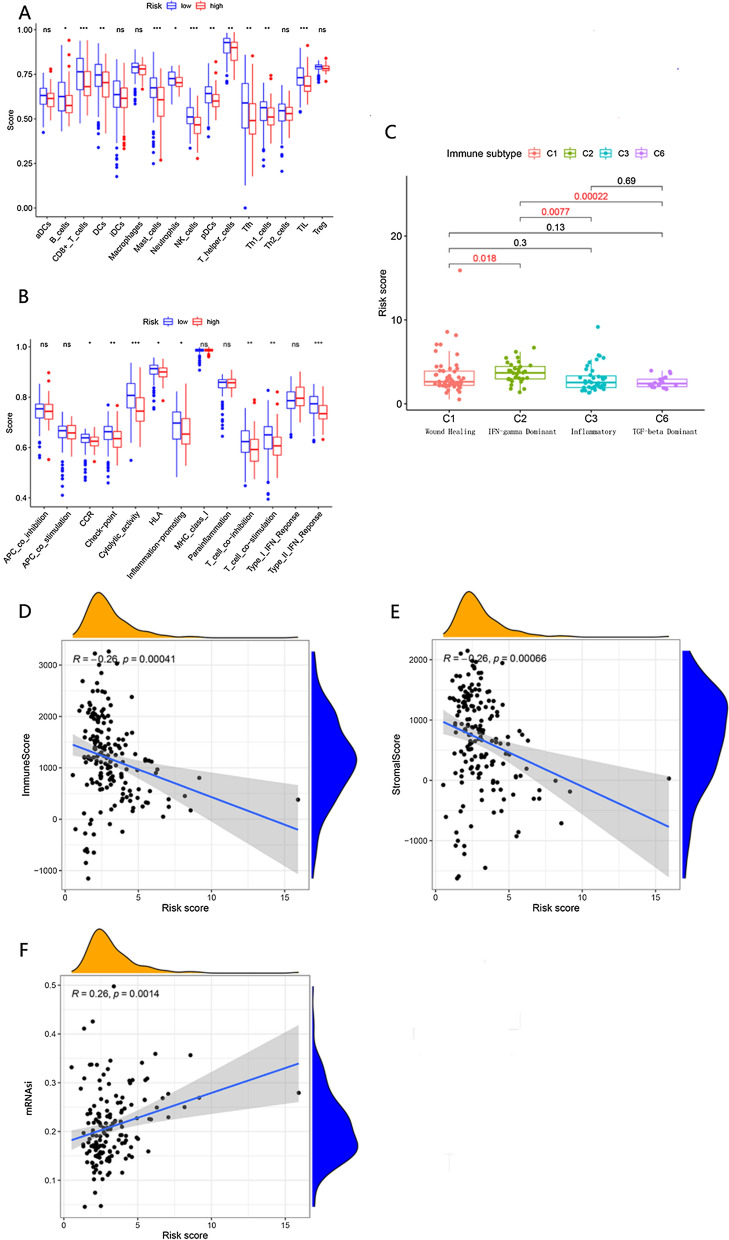
Correlation of YOD1 expression with immune infiltration level and correlation analysis of stemness index in PAAD. **A** YOD1 expression is significantly negatively related to infiltrating levels of CD8 + T cells, mast cells, NK cells and TIL cells in PAAD (*Indicate that the results are statistically significant). **B** YOD1 expression has significant negative correlations with infiltrating levels of cytolytic activity cells and type II IFN response cells in PAAD (*Indicate that the results are statistically significant). **C** The expression level of YOD1 was significantly different in C2 immunosubtypes compared with C1, C3 and C6 immunosubtypes. **D**, **E** YOD1 expression has negative significant correlations with Immune cells and stromal cells infiltrating levels in PAAD. **F** mRNA level of YOD1 is significantly positively related to stemness index (mRNAsi)

To determine whether the expression of YOD1 affects the composition of the PAAD immune microenvironment, we used single-sample gene set enrichment analysis (ssGSEA). The results in Fig. 5A showed that the high expression of YOD1 was closely related to the decreased infiltration of tumor-infiltrating lymphocytes (TIL) (*P* < 0.01), CD8 + T cells (*P* < 0.01), DCs (*P* < 0.01), and NK cells (*P* < 0.01). The decrease of tumor killer cells will facilitate the immune escape of tumor cells. In immune cell function, the high expression of YOD1 is closely related to the reduction of type II interferon response (Fig. 5B). In the immune subtypes, the high expression of YOD1 is closely related to IFN-γ dominant, which is statistically different from other subtypes (Fig. 5C).

To further confirm whether the level of YOD1 expression affects the immune microenvironment of PAAD, we used the ESTIMATE algorithm to calculate the immune cell score and stromal cell score [20]. Immune cells and stromal cells are the two main non-tumor components. The higher the immune score and stromal score are, the higher the degree of non-tumor cell infiltration in tumor tissue is. Immune cell score (R = − 0.26, *P* = 0.00041) and stromal cell score (R = − 0.26, *P* = 0.00066) showed a negative correlation with the expression of YOD1 (Fig. 5D, E). It was speculated that when YOD1 was overexpressed, the tumor purity was higher.

Cancer stem cells (CSCs) share common biological characteristics with adult stem cells, such as lifespan, self-renewal ability, differentiation, and drug resistance, which play a decisive role in cancer progression [23]. Some studies have found that pancreatic cancer stem cells are highly drug-resistant cells that preferentially drive tumorigenesis and development, and these are associated with poor prognosis of PAAD patients [24]. The results showed that the expression of YOD1 was positively correlated with mRNAsi, R = 0.26 (*P* = 0.00014) (Fig. 5F). It is suggested that the increased expression of YOD1 is related to the increase of stem cell characteristics. Besides, it promotes the occurrence and development of tumor.

### Enrichment analysis of YOD1

**Fig. 6:**
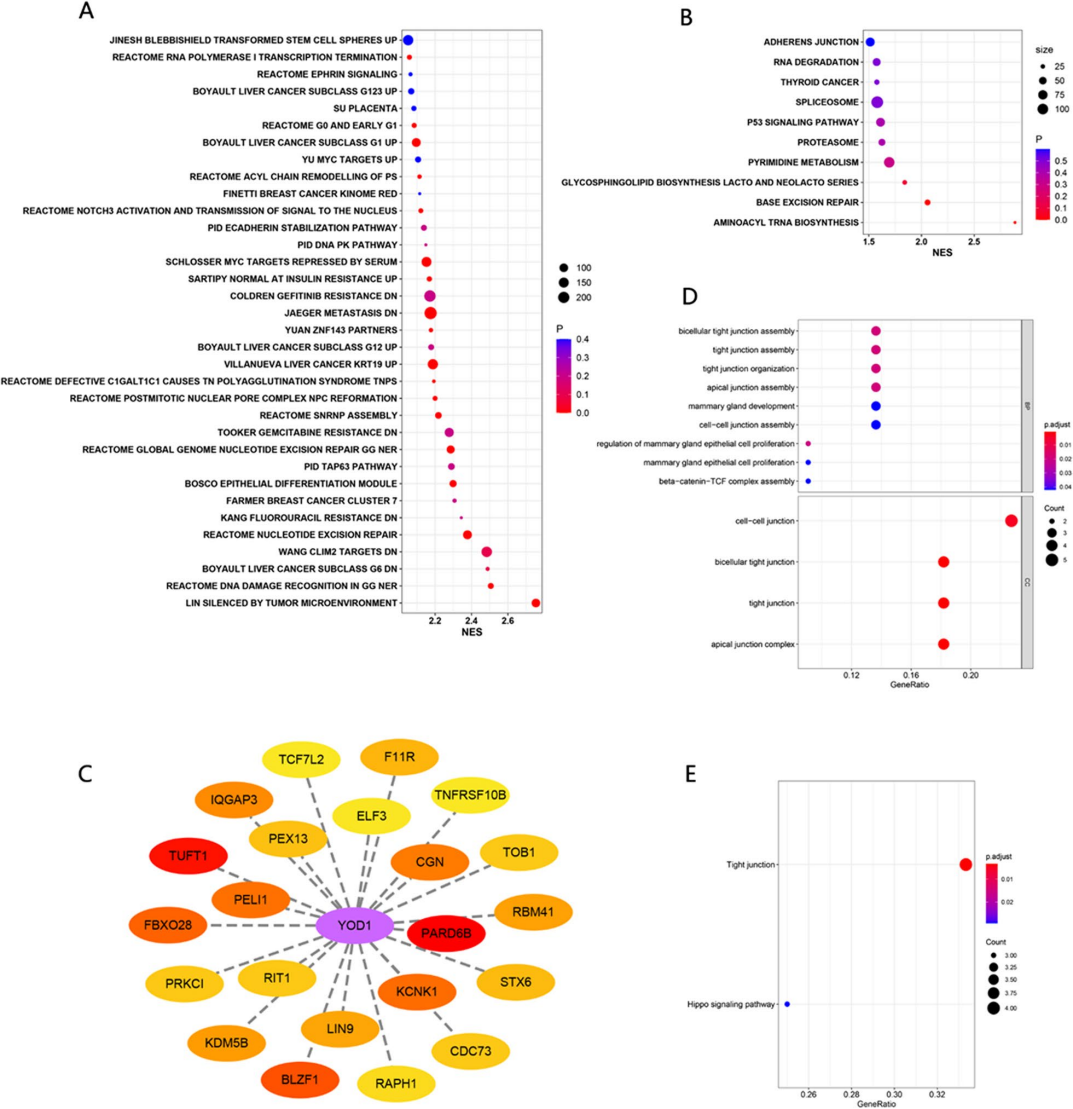
YOD1 co-expression network. **A** GO enrichment gene sets of YOD1. **B** KEGG enrichment gene sets of YOD1. **C** YOD1-related protein protein interaction network (PPI). **D** GO enrichment gene sets of YOD1 co-expression proteins. **E** KEGG enrichment gene sets of YOD1 co-expression proteins

After understanding the prognostic role of YOD1 in PAAD, we will explore the biological function of YOD1 in PAAD. GO and KEGG enrichment results were displayed in the first 30 bits of NES by bubble diagram. GO analysis showed that the high expression of YOD1 was correlated with biological functions such as metastasis, gemcitabine resistance and transcription (Fig. 6A). KEGG pathway analysis showed that the high expression of YOD1 was correlated with other pathways such as base excise and repair, p53 signaling pathway, RNA degradation, cell–cell adhesion and connection (Fig. 6B). GSEA v.4.0 c2.all.v7.4.symbols.gmt [Curated] and c2.cp.kegg. v7.4.symbols.gmt [Curated] gene sets were analyzed, which satisfied the *P-*value < 0.05 and false discovery rate (FDR) < 0.25. As shown in Additional file 1: Fig. S3, the high expression of YOD1 in PAAD is highly correlated with adhesion, metastasis, invasion, Gefitinib chemotherapy resistance, DNA repair, tumor hypoxic microenvironment and p53, Notch, NRG1, MYC, EGFR, Hippo signaling pathway.

Linkedomics analysis found that 6,505 genes were significantly associated with YOD1 in PAAD(*P* < 0.05,FDR < 0.01). There were 2968 positively correlated genes and 3537 negatively correlated genes. The first 50 positive and negative co-expressed genes are shown in the heat map. Among genes correlated with YOD1, 23 genes were associated with OS (*P* < 0.05), while 33 genes were negatively correlated with OS (*P* < 0.05) (Additional file 1: Fig. S4).

We used the "clusterProfiler" R package to perform PPI, GO and KEGG enrichment analysis on 23 positively related genes, as shown in Fig. 6C–E. The discovery of the enrichment of cell-related connections and the Hippo pathway is consistent with our previous results. It is proved that YOD1 may regulate the occurrence, development and prognosis of pancreatic cancer through these two key aspects.

**Fig. 7:**
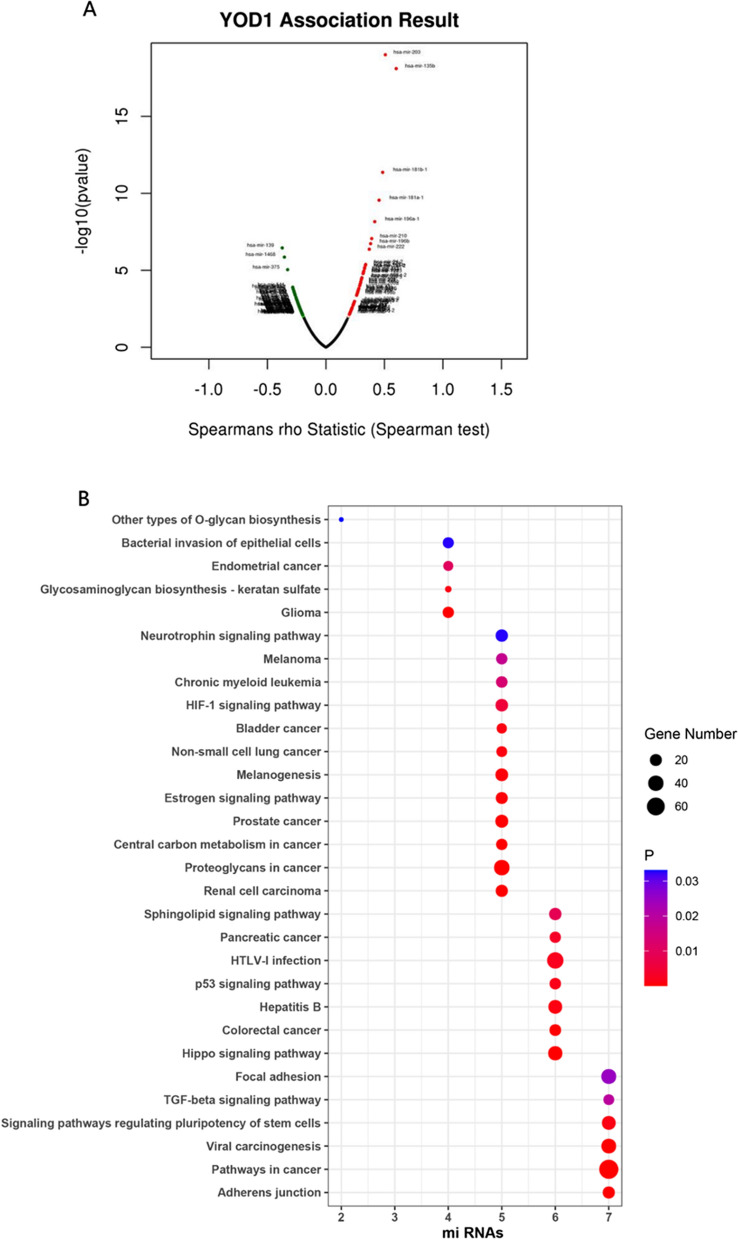
miRNA-YOD1 co-expression network **A** miRNA-YOD1 Volcano plot. **B** Enrichment gene sets of miRNAs which negatively regulate YOD1

To identify upstream molecules that regulate YOD1 mRNA levels in PAAD, we evaluated the miRNA-YOD1 network in PAAD. The result is shown in Fig. 7A. Usually miRNAs inhibit the expression of target genes, so we focused on negative correlations and *P* < 0.0005 of 12 miRNAs (hsa-mir-675; hsa-miR-150; hsa-miR-202; hsa-miR-1468; hsa-miR-139; hsa-miR-140; hsa-miR-218; hsa-miR-9; hsa-miR-342; hsa-miR-375; hsa-miR-500a; hsa-miR-433). Analysis of the above 13 miRNAs showed that, as shown in Fig. 7B, they could similarly focus on cancer, cell adhesion, p53, TGF-β, Hippo and other cancer-related signaling pathways. And among the above 12 miRNAs, we found that hsa-miR-1468; hsa-miR-139; hsa-miR-140; hsa-miR-218; hsa-miR-9; hsa-miR-342; hsa-miR-375; hsa-miR-500a were negatively associated with poor overall survival of PAAD (Additional file 1: Fig. S5 and Table 3). Therefore, these miRNAs may affect the prognosis of PAAD by regulating the expression of YOD1.

**Table 3:** Overall survival of miRNA that target YOD1 in PAAD

miRNA	Low expression	High expression	Prognose	*P*-value	HR
hsa-mir-675	n = 62	n = 116	N/A	0.053	1.54 (0.99–2.4)
hsa-mir-150	n = 83	n = 95	N/A	0.095	0.71 (0.47–1.06)
hsa-mir-202	n = 50	n = 128	N/A	0.19	0.75 (0.49–1.16)
hsa-mir-1468	n = 118	n = 60	Favorable	0.02	0.59 (0.37–0.92)
hsa-mir-139	n = 133	n = 45	Favorable	0.0047	0.46 (0.26–0.8)
hsa-mir-140	n = 96	n = 82	Favorable	0.017	0.6 (0.39–0.92)
hsa-mir-218	n = 50	n = 128	Favorable	0.0038	0.53 (0.35–0.82)
hsa-mir-9	n = 119	n = 59	Favorable	0.038	0.61 (0.39–0.98)
hsa-mir-342	n = 119	n = 59	Favorable	0.0013	0.45 (0.27–0.74)
hsa-mir-375	n = 93	n = 85	Favorable	0.012	0.59 (0.39–0.9)
hsa-mir-500a	n = 84	n = 94	Favorable	0.0088	0.58 (0.39–0.88)
hsa-mir-433	n = 116	n = 62	N/A	0.069	0.66 (0.43–1.04)

### Function verification of YOD1

**Fig. 8:**
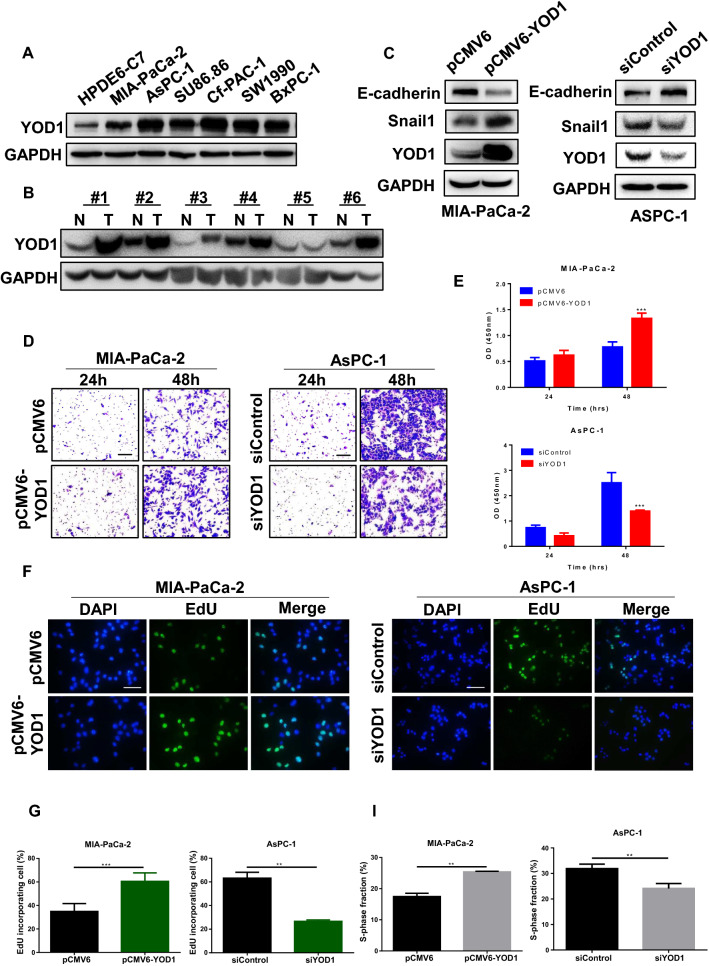

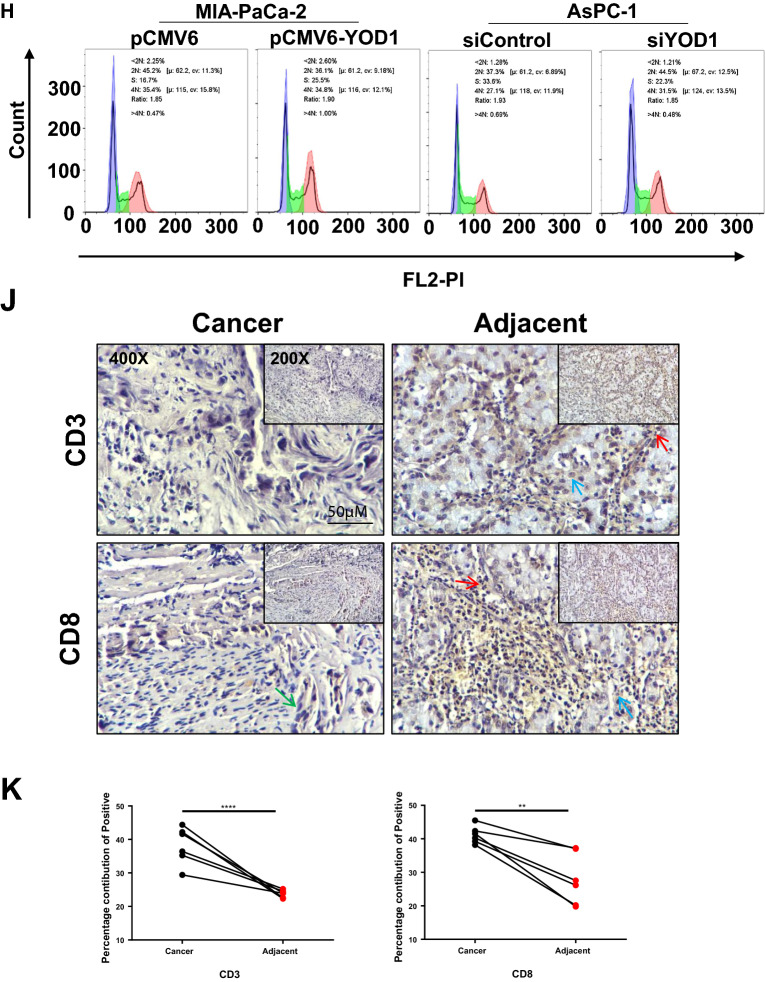
YOD1 promotes the proliferation and metastasis of pancreatic cancer cells. **A** The expression of YOD1 in cancer tissues and adjacent cancer tissues of seven groups of clinical samples. N, normal; T, tumor. **B** The expression of YOD1 between six types of pancreatic cancer cells and normal pancreatic cells (HPDE6-C7). **C** EMT changes of AsPC-1 and MIA-PaCa-2 cells with YOD1 knockdown or YOD1- overexpression. **D**, **E** Transwell assays of 24 h and 48 h were performed to detect the metastasis in AsPC-1 and MIA-PaCa-2 cells with YOD1- knockdown or YOD1- overexpression. Scale bar, 100 μm. **F**, **G** EdU incorporation assays were performed to detectcell proliferation in AsPC-1 and MIA-PaCa-2 cells with YOD1- knockdown or YOD1- overexpression. Scale bar, 50 μm. **H**, **I** Flow Cytometry was performed to detect cell cycle in AsPC-1 and MIA-PaCa-2 cells with YOD1-knockdown or YOD1-overexpression, and the S-phase fraction reflects cell proliferation. **J**, **K** IHC was performed to detect CD3 and CD8 expression in cancer and normal tissues. Scale bar, 50 μm, 400× magnification. The upper right small image is magnified 200× . Blue for pancreatic cells, red for lymphocytes and green for cancer cells

To verify the function of YOD1, we first compared the expression levels of YOD1 in 6 PAAD cell lines with normal pancreatic cells. It was found that the expression of YOD1 in 6 PAAD cells was higher than normal pancreatic cells HPDE6-C7 (Fig. 8A). Further, we compared the expression of YOD1 protein in the cancerous tissues with the adjacent tissues more than 5 cm away from the cancerous tissues of the six pancreatic cancer patients by Western blot and IHC experiments, and we found that the expression of YOD1 in the cancerous tissues was higher than that in the adjacent tissues (Fig. 8B, Additional file 1: Fig. S6A).

We selected MIA-PaCa-2 with low YOD1 expression to express YOD1 highly, and metastatic malignant PAAD with high YOD1 expression AsPC-1 to knock down YOD1 for the experiment. Transient transfection was performed on the cells to verify the expression of EMT-related factors (Fig. 8C)**,** and transwelled for 6 h to exclude the effect of proliferation on metastasis (Additional file 1: Fig. S6B, C) and time-dependent Transwell experiments to mimic tumor metastasis (Fig. 8D, E). Changes in the protein levels of epithelial-mesenchymal transition (EMT) markers, including E-cadherin and Snail1, were consistent with the results of Transwell experiment. All the above indicated that after YOD1 knockdown, the migration ability of AsPC-1 cells was decreased, while the high expression of YOD1 increased the migration ability of MIA-PaCa-2 cells. EdU (5-ethynyl-2′-deoxyuridine) was a thymidine analog with an alkyne group rarely found in natural compounds that can replace thymine (T) during DNA replication Infiltrating into the synthesizing DNA molecule. The DNA replication activity can be directly and accurately detected based on the specific reaction between Apollo fluorescent dye and EdU, which can be used to detect cell proliferation (Fig. 8F, G). The cell cycle was detected by Flow Cytometry, and cell proliferation was judged by the ratio of cells in S phase (Fig. 8H, I). The results showed that YOD1 was decreased, and the activity and proliferation of AsPC-1 cells were decreased, while YOD1 was increased, and the activity and proliferation of MIA-PaCa-2 cells were increased. IHC experiments were performed on the cancer tissues of 6 pancreatic cancer patients and the adjacent tissues more than 5 cm away from the cancer tissues to detect CD3 and CD8 which reflect the tumor immune microenvironment. We found that total mature T cells and cytotoxic T lymphocytes were reduced, indicating that YOD1 may be able to reduce the ability of human body immune to kill tumors (Fig. 8J).

In conclusion, the experiments were consistent with the previous bioinformatics analysis results, and YOD1 may act as a proto-oncogene and have certain effects on the proliferation and metastasis of PAAD cells. At the same time, expressing level of YOD1 is related to degree of tumor immune microenvironment.

## Discussion

PAAD is a malignancy with a poor prognosis, with a 5-year survival rate of less than 9%. PAAD is difficult to diagnose early, usually at an advanced stage, and less than 20% of patients are eligible for radical resection [25]. Therefore, identification of novel prognostic and diagnostic biomarkers for early diagnosis of cancer may help improve the prognosis of patients with PAAD. Despite recent advances in the molecular mechanism of PAAD, the molecular mechanism of PAAD carcinogenesis and development remains elusive.

Ubiquitination is an important post-translational modification mechanism that regulates many biological processes. In recent years, more and more attention has been paid to the process of ubiquitination. Some researchers have demonstrated that ubiquitination plays an important role in the formation and development of various tumors, but few studies have been conducted in PAAD. Therefore, we selected OTUD family, a subgene family with editing deubiquitination enzyme and strong ubiquitin-specific recognition, to carry out the research on the molecular mechanism of PAAD through a variety of public databases and joint basic experiments. In this study, we first studied the mRNA levels of 8 genes from the OTUD family in PAAD and normal tissues. Analysis of multiple data sets showed that YOD1 was highly expressed in patients with PAAD. ROC results showed that YOD1 had good diagnostic performance. In order to further study the prognostic value of YOD1 gene, we constructed a risk model based on the expression level of YOD1 mRNA, plotted the risk curve using Kaplan–Meier plotter, and verified the risk curve using COX regression analysis. The results showed that high expression of YOD1 was an independent factor affecting the prognosis of PAAD patients.

In addition, an increasing number of research has found that tumor-infiltrating lymphocytes, especially CD8 + cells, affect patient outcomes, chemotherapy and immune efficacy [26]. Another important aspect of this study is that YOD1 expression is associated with different levels of immune infiltration in PAAD. The tumor-infiltrating lymphocytes were significantly reduced in the high expression group, and the immune and matrix scores were lower as calculated by the ESTIMATE algorithm. Together, these findings suggest that YOD1 plays an important role in the recruitment and regulation of immune-infiltrating cells in PAAD. Moreover, it has been demonstrated that the expression of dominant negative YOD1 transgenes (YOD1-C160s) leads to enhanced function, facilitates the cross-presentation of exogenous antigens by Antigen presenting cells (APCs), and enhances CD8 + T cell response in vitro and in vivo [27].

Next, we investigated the potential signaling pathway of YOD1 in PAAD by GSEA and Linkedomic functional enrichment analysis. Analysis suggested that YOD1 was related to intercellular adhesion, p53, Hippo pathway, metastasis, drug resistance, etc. Imbalanced intercellular adhesion is characteristic of many pathological conditions that can lead to increased tumor invasion and metastasis. The enrichment of YOD1 co-expressed genes also enriches the Hippo pathway. Studies have confirmed that under high cell density, the progression of liver cancer can be inhibited by the miR-21 − YOD1 − ITCH − LATS signal axis [28]. Youngeun Kim et al. also proved that YOD1 is a potent activator of YAP in liver cancer [29]. The Hippo pathway plays a vital role in many cancers including pancreatic cancer. Studies have shown that miRNA-10a inhibits the Hippo signaling pathway by inhibiting WWC2 expression, thereby reducing stem maintenance and EMT in PAAD [30]. In addition, p53 signaling pathway is related to the regulation of cell cycle, cell senescence and apoptosis. In PAAD, it has been reported that the downregulation of CCNB1 gene activates p53 signaling pathway, thus inhibiting the proliferation of PAAD cells and promoting the senescence of PAAD cells [31]. For TGF-β signaling, it can initiate SMAD signaling pathways and non-SMAD signaling pathways. Due to the balance change between the above, TGF-β often changes from tumor suppression factor to tumor promotion factor during cancer progression [32]. Studies have reported that YOD1 RNAi in Human Oral Keratinocytes can inhibit cell proliferation and migration related to the pathogenesis of nonsyndromic cleft lip with or without cleft palate (NSCL/P) through TGF-β 3 signaling [33], which suggested that the targeted inhibition of YOD1 in the advanced stage of cancer may also play a role in inhibiting cell proliferation and migration. The above may be the key pathways that YOD1 promotes the occurrence and development of pancreatic cancer and affect the prognosis.

The miRNA usually acts as upstream molecules to negatively regulate gene expression, thus affecting the occurrence and development of diseases. Recent studies [34] have shown that miRNAs play an important role in the occurrence of cancer, including PAAD. However, miRNAs that regulate YOD1 expression in PAAD are unknown. It has been reported that miR-4429 inhibits the malignant development of ovarian cancer by targeting YOD1. Yan R et al. found that dexthasone therapy can inhibit the expression of YOD1 by upregulation of miR-520a-3p, thereby inhibiting the cellular malignancies of osteosarcoma [35]. In this study, we enriched 12 miRNAs that were significantly negatively correlated with YOD1. Subsequent studies found that these miRNAs were significantly enriched in cancer, cell adhesion, p53, TGF-β, Hippo and other cancer-related signaling pathways. Eight of the miRNAs were significantly correlated with OS of PAAD, possibly by inhibiting YOD1. miR-139, miR-218 in these miRNAs affecting prognosis. It has been shown [36] that miR-139 has obvious inhibition effect on the growth, migration and invasion of pancreatic cancer cells, and miR-218 is involved in forming a signal axis affecting the development of pancreatic cancer.This is consistent with the results of this study.

To further evaluate the effect of YOD1 overexpression on the proliferation, migration and immune microenvironment of PAAD cells. Firstly, Western blot was used to detect the clinical samples of 7 groups of patients from the First Affiliated Hospital of China Medical University. On the whole, compared with the adjacent tissues, the expression of YOD1 in cancer tissues was high. Then the expression of YOD1 in normal pancreatic cells was compared with that in 6 PAAD cells. Consistent results showed that the expression of YOD1 in PAAD cells was higher than that in normal pancreatic cells. We selected MIA-PaCa-2, a pancreatic cancer cell with low YOD1 expression, and AsPC-1, a metastatic pancreatic cell with a high degree of malignancy. YOD1 was highly expressed in MIA-PaCa-2 cells, and YOD1 was knocked down in AsPC-1 cells. The results of Transwell and Western blot of EMT-related factors proved that the cells with high YOD1 expression in PAAD had a stronger migration. EDU and Flow Cytometry showed that high expression of YOD1 could significantly promote the proliferation of PAAD cells. In previous studies, the high expression of YOD1 has been confirmed to be positively correlated with the proliferation of hepatocytes [28] and the migration of human oral keratinocytes, while inhibiting the proliferation of cervical cancer cells [37], which may be related to the different cell morphology in different organs. We also found that YOD1 may be closely related to the tumor immune microenvironment by IHC experiments. Some researchers found that immunosuppressive condition of tumor microenvironment influence effect of immune checkpoint blockade. Especially the presence or absence of CD8^+^ T cells greatly affects the sensitivity to PD-1 immunoblockade therapy [38]. In our study, Overexpression of YOD1 was associated with decreased CD8^+^ T cells expression. It maybe can be speculated that YOD1 can serve as a new way to improve the effectiveness of pancreatic cancer immunotherapy.

## Conclusion

The analysis results from the public data and our own experimental results demonstrate that YOD1 or OTUD2 may serve as a biomarker for the diagnosis and prognosis of PAAD. The expression of YOD1 affects the proportion of immune cell immersion in the micro-environment of pancreatic tumor and they have potential therapeutic value. p53, TGF-β and especially Hippo signaling pathways may be the key pathways of YOD1 regulation. Further experimental validation should be performed to demonstrate the biological effects of YOD1. Above, we provided a preliminary basis for evaluating the prognosis of PAAD patients, exploring potential therapeutic targets and predicting the efficacy of treatment.

## Supplementary Information

**Additional file 1: Figure S1.** Differential expression of OTUD family members in pancreatic normal tissue, primary tumors and cancerous pancreatic cell lines. A–G Boxplots graphs show relative expression of OTUD1, YOD1, OTUD3, OTUD4, OTUD5, OTUD6B and ALG13in dataset of MERAV. **Figure S2.** The expression of OTUD family members in PAAD patients (GEPIA). A-G Box plots derived from gene expression data for GEPIA comparing the expression of OTUD1, YOD1, OTUD3, OTUD4, OTUD5, OTUD6B, and ALG13 in cancer tissue and normal tissues; the p-value was set at 0.05. *Indicate that the results are statistically significant. **Figure S3.** GSEA analysis of of YOD1-related enrichment gene sets. **Figure S4.** Linkedomics analysis of YOD1 positive correlation and negative correlation gene expression (A) Heat map of YOD1 positive correlation gene expression. (B) 23 positive correlation genes were associated with OS (P<0.05). (C) Heat map of YOD1 negative correlation gene expression. (D) 33 negative correlation genes were associated with OS (P<0.05). **Figure S5.** Survival curves comparing patients with high (red) and low (black) miRNAs expression which were associated with OS in PAAD were plotted using Kaplan–Meier plotter database. **Figure S6.** (A) IHC was performed to detect YOD1 expression in cancer and normal tissues. Scale bar, 50μm, 400× magnification. (B, C) Transwell assays of 6 h were performed to detect the metastasis in AsPC-1 and MIA-PaCa-2 cells with YOD1- knockdown or YOD1- overexpression. Scale bar, 100μm.

**Additional file 2: Table S1.** 6 patients clinical information. PASC, pancreatic adenosquamous carcinoma; PAAD, pancreatic adenocarcinoma; PMN, pancreatic mucinous neoplasm; T, tumor; N, node; M, metastasis.

## Data Availability

The datasets generated and analysed during the current study are available in the public databases TCGA database (http://www.cancer.gov/tcga), GEO database (https://www.ncbi.nlm.nih.gov/gds/) with the accession numbers: GSE15471, GSE16515, GSE28735, GSE71729, and GSE62165, GTEx database (https://gtexportal.org/), ONCOMINE database (www.oncomine.org), HPA (https://www.proteinatlas.org) and cBioPortal (www.cbioportal.org).

## Abbreviations

CSCs: Cancer stem cells
CTC: Circulating tumor cells
DC: Dendritic cells
DFS: Disease Free Survival
DUBs: Deubiquitinating enzymes
Edu: 5-Ethynyl-2′-deoxyuridine
ER: Endoplasmic reticulum
FDR: False detection rate
GO: Gene Ontology
GSEA: Gene set enrichment analysis
HPA: Human Protein Atls
HR: Hazard ratio
IHC: Immunohistochemistry
KEGG: Kyoto Encyclopedia of Genes and Genomes
MERAV: Metabolic Generapid Visualizer
OCLR: One-class logistic regression
OS: Overall Survival
OTU: Ovarian tumor-associated protease
PAAD: Pancreatic adenocarcinoma
PASC: Pancreatic adenosquamous carcinoma
PC: Pancreatic cancer
PDAC: Pancreatic ductal adenocarcinoma
PMN: Pancreatic mucinous neoplasm
RFS: Relapse-free survival
ROC: Receiver operating characteristic
SDS-PAGE: SDS–polyacrylamide gel electrophoresis
ssGSEA: Single-sample gene set enrichment analysis
TCGA: The Cancer Genome Atlas
TIL: Tumor-infiltrating lymphocytes
Ub: Ubiquitin
