# Supplementary material for: YOD1 serves as a potential prognostic biomarker for pancreatic cancer
Source: Cancer Cell Int. 2022 May 31;22:203. doi: 10.1186/s12935-022-02616-9 (PMC9158148; doi:10.1186/s12935-022-02616-9)
Supplement: Supplementary file 1 — Additional file 1: Figure S1. Differential expression of OTUD family members in pancreatic normal tissue, primary tumors and cancerous pancreatic cell lines. A–G Boxplots graphs show relative expression of OTUD1, YOD1, OTUD3, OTUD4, OTUD5, OTUD6B and ALG13in dataset of MERAV. Figure S2. The expression of OTUD family members in PAAD patients (GEPIA). A-G Box plots derived from gene expression data for GEPIA comparing the expression of OTUD1, YOD1, OTUD3, OTUD4, OTUD5, OTUD6B, and ALG13 in cancer tissue and normal tissues; the p-value was set at 0.05. *Indicate that the results are statistically significant. Figure S3. GSEA analysis of of YOD1-related enrichment gene sets. Figure S4. Linkedomics analysis of YOD1 positive correlation and negative correlation gene expression (A) Heat map of YOD1 positive correlation gene expression. (B) 23 positive correlation genes were associated with OS (P<0.05). (C) Heat map of YOD1 negative correlation gene expression. (D) 33 negative correlation genes were associated with OS (P<0.05). Figure S5. Survival curves comparing patients with high (red) and low (black) miRNAs expression which were associated with OS in PAAD were plotted using Kaplan–Meier plotter database. Figure S6. (A) IHC was performed to detect YOD1 expression in cancer and normal tissues. Scale bar, 50μm, 400× magnification. (B, C) Transwell assays of 6 h were performed to detect the metastasis in AsPC-1 and MIA-PaCa-2 cells with YOD1- knockdown or YOD1- overexpression. Scale bar, 100μm. [file 12935_2022_2616_MOESM1_ESM.pptx]

## Slide 1
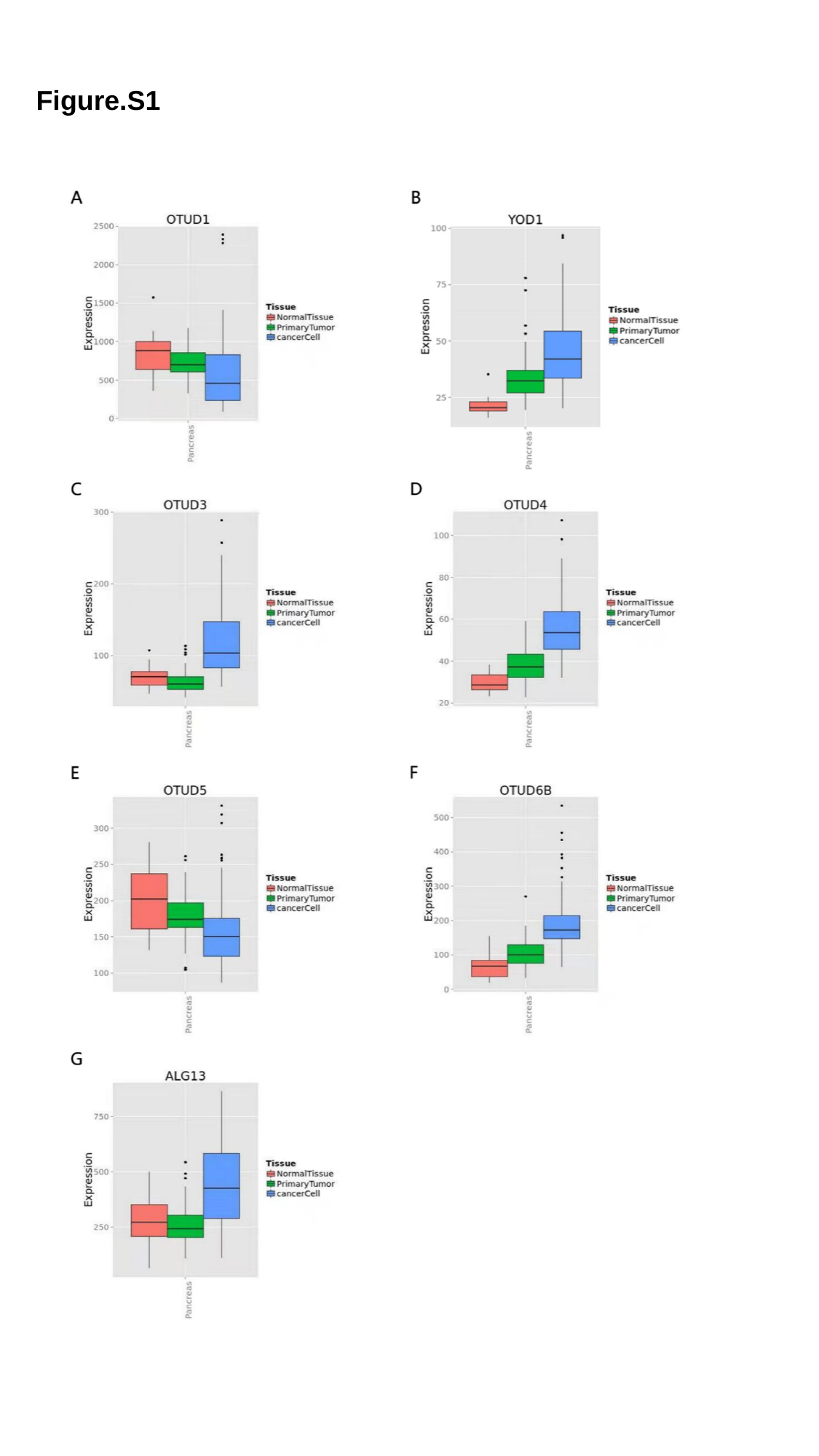

Figure.S1

## Slide 2
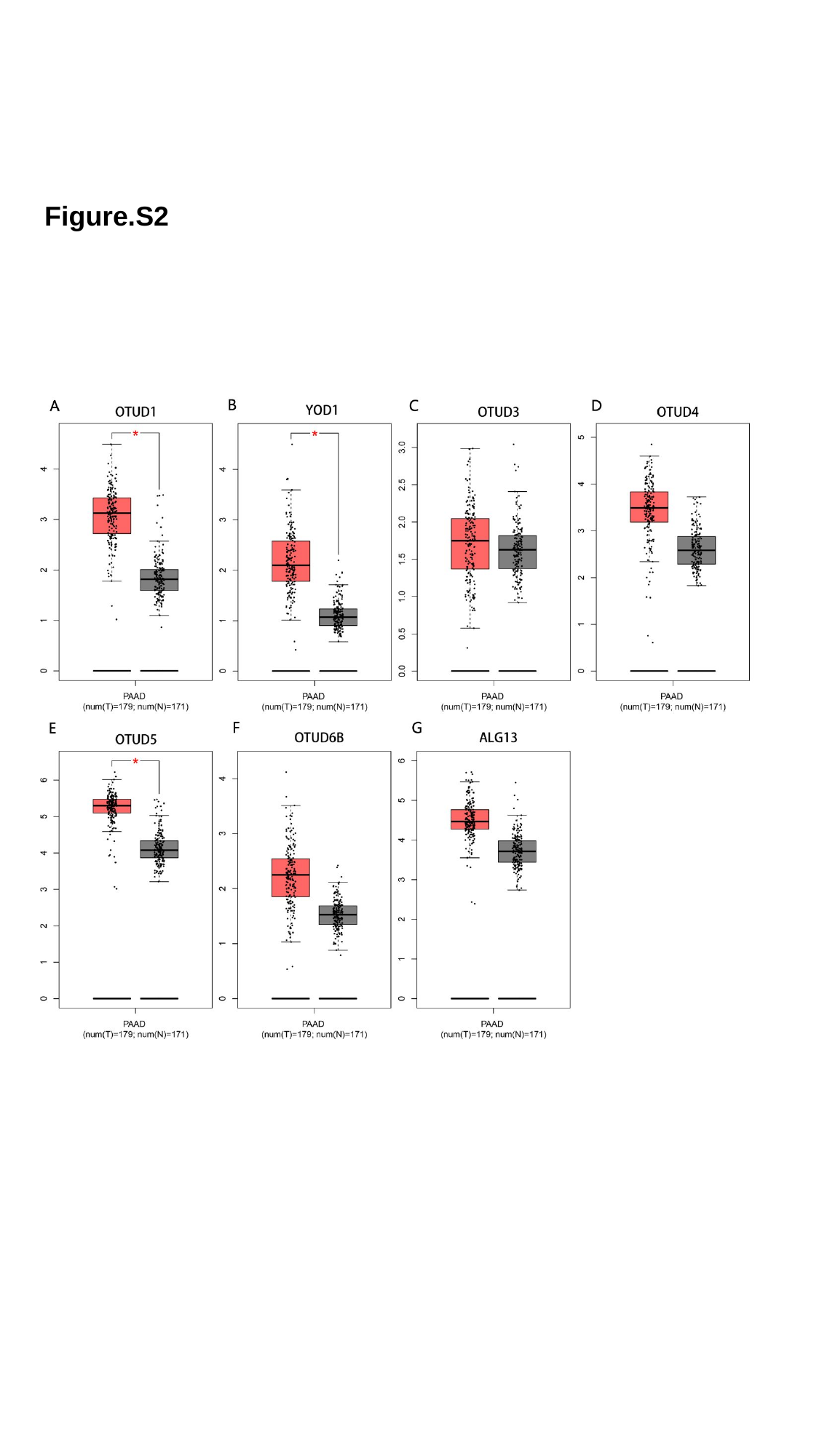

Figure.S2

## Slide 3
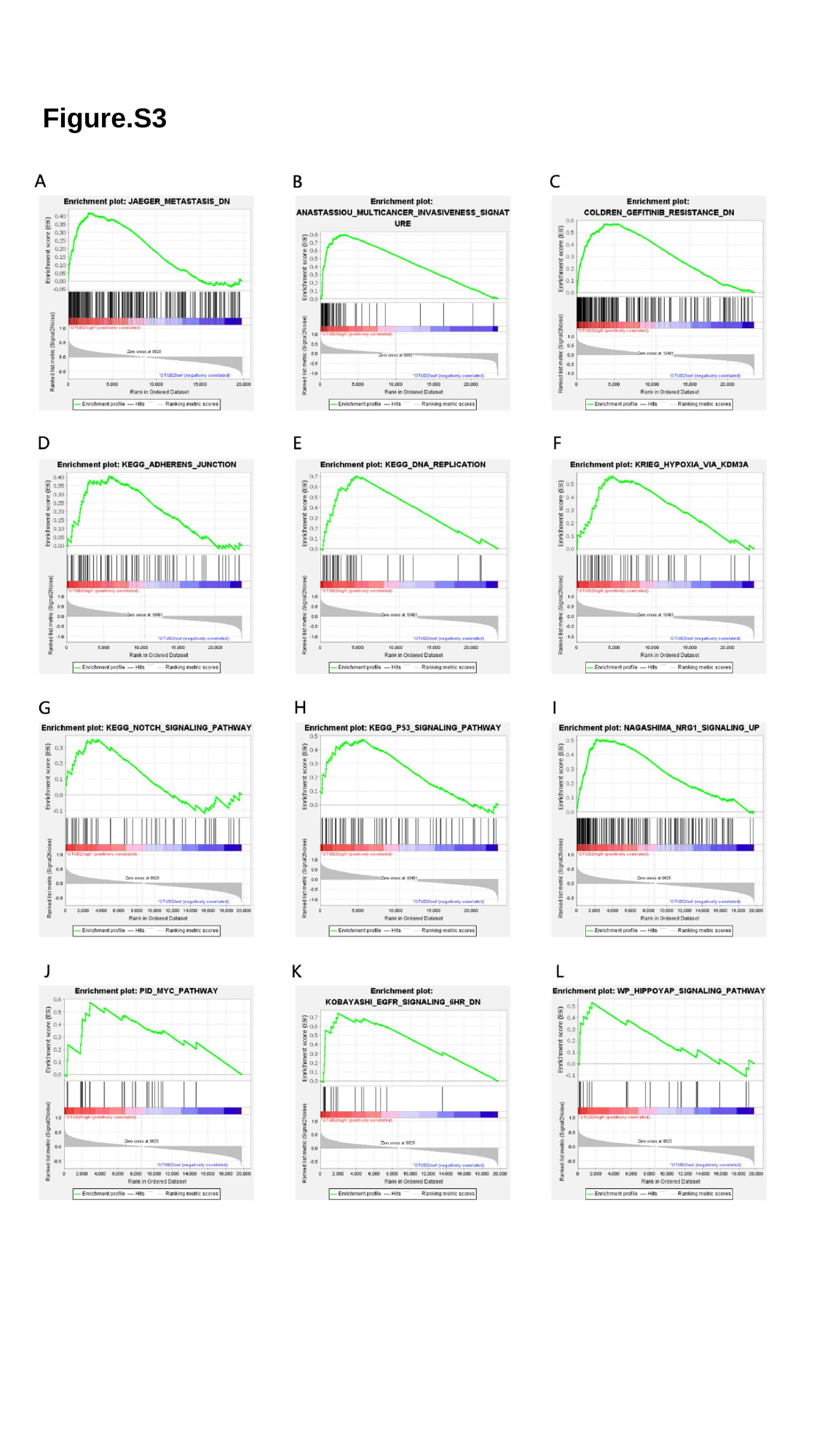

Figure.S3

## Slide 4
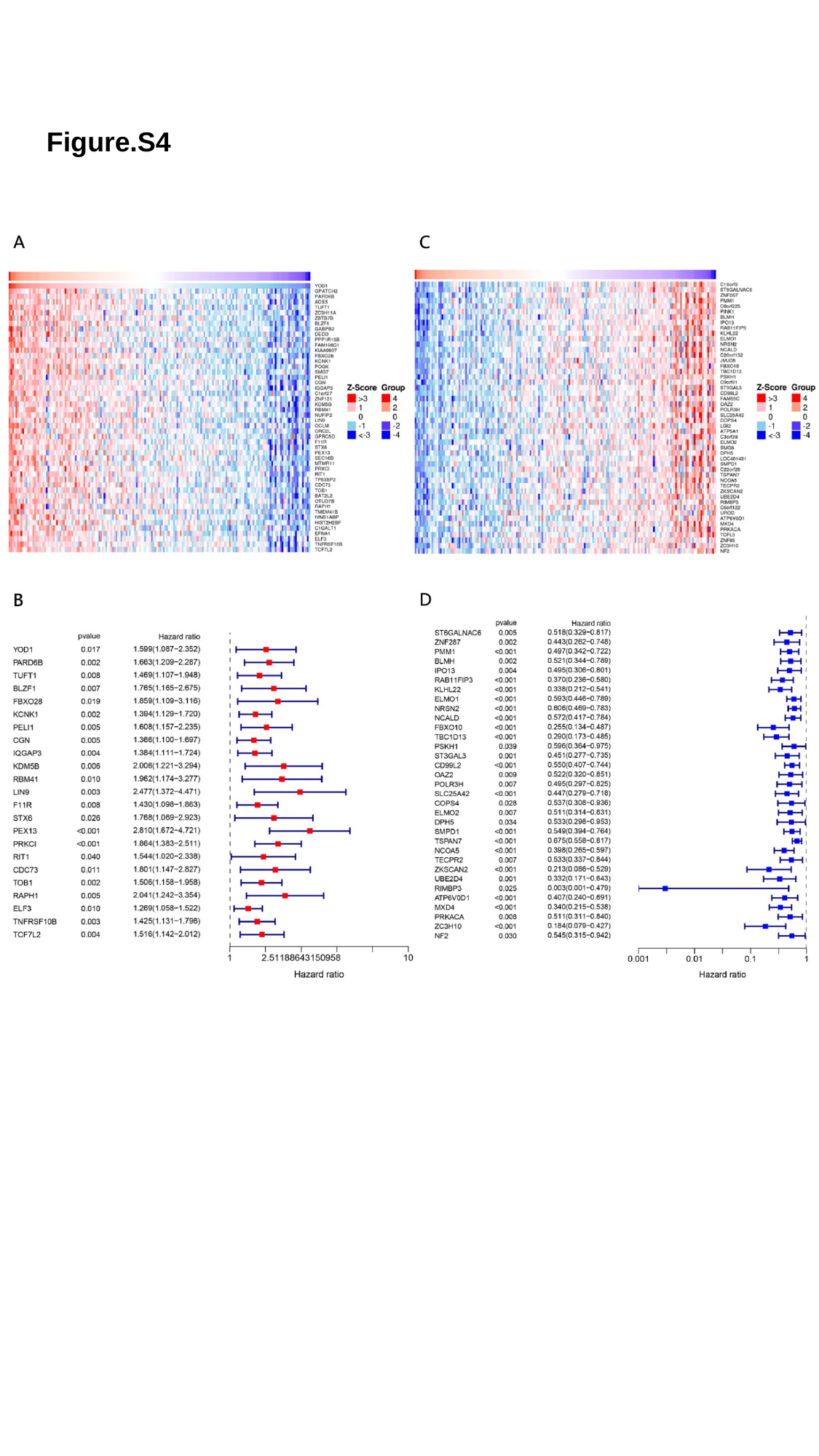

Figure.S4

## Slide 5
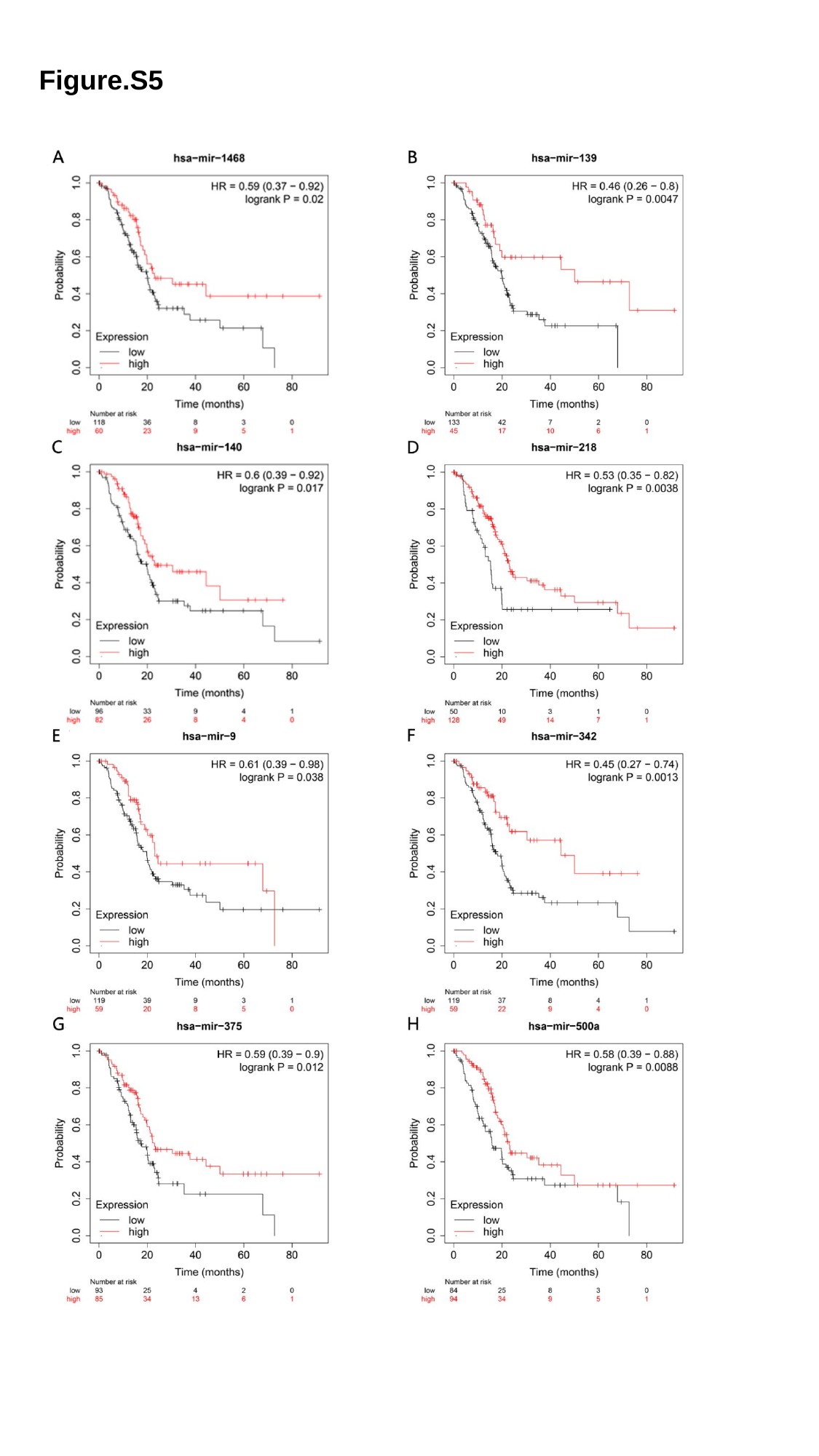

Figure.S5

## Slide 6
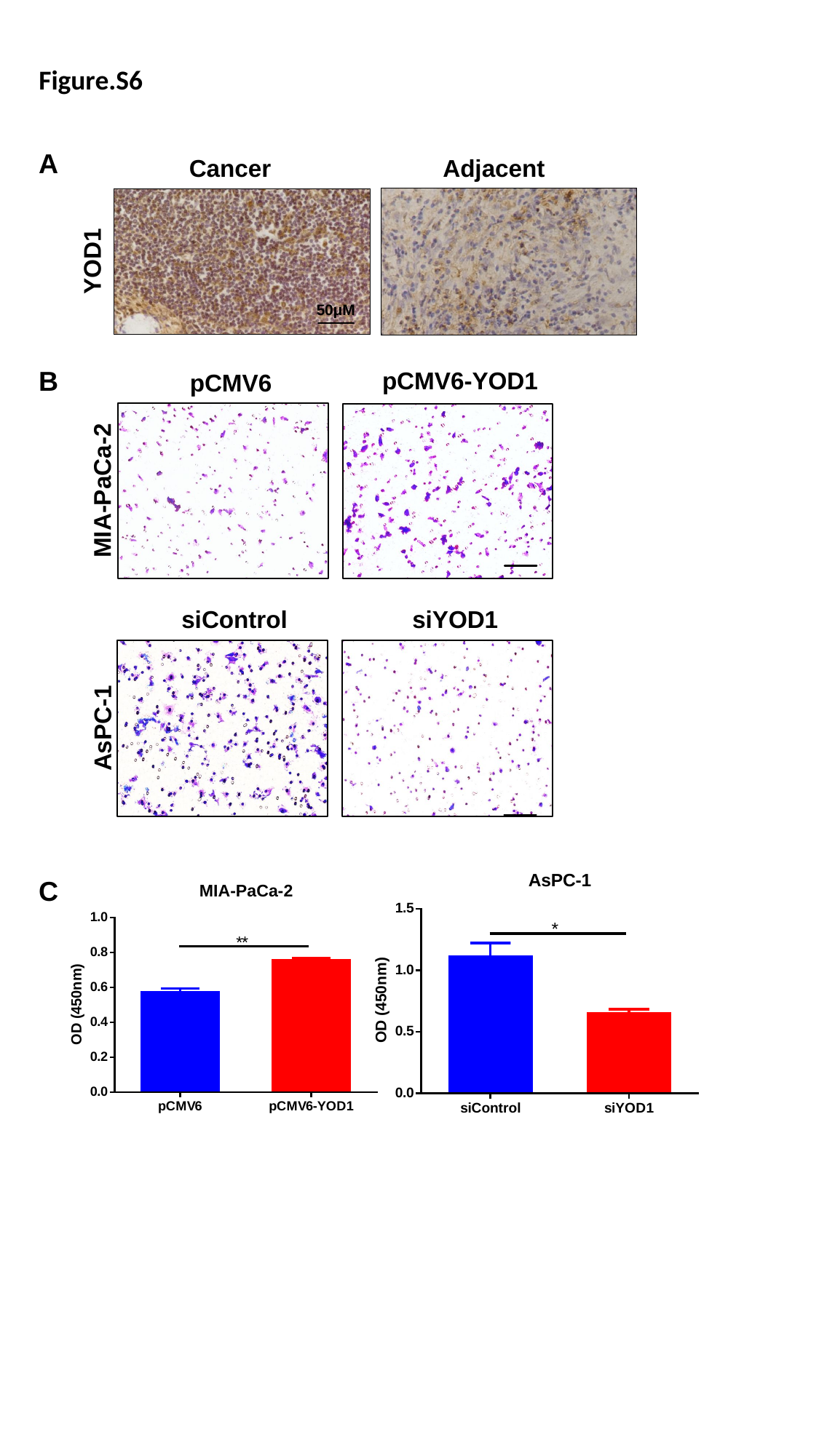

Figure.S6
A
Cancer
Adjacent
YOD1
50μM
B
pCMV6-YOD1
pCMV6
MIA-PaCa-2
siControl
siYOD1
AsPC-1
C
