# Supplementary material for: YOD1 serves as a potential prognostic biomarker for pancreatic cancer
Source: Cancer Cell Int. 2022 May 31;22:203. doi: 10.1186/s12935-022-02616-9 (PMC9158148; doi:10.1186/s12935-022-02616-9)
Supplement: Supplementary file 2 — Additional file 2: Table S1. 6 patients clinical information. PASC, pancreatic adenosquamous carcinoma; PAAD, pancreatic adenocarcinoma; PMN, pancreatic mucinous neoplasm; T, tumor; N, node; M, metastasis. [file 12935_2022_2616_MOESM2_ESM.docx]

Table.S1 6 patients clinical information

| Number | Gender | Age | Pathology | Tumor size (mm) | Lymph Node Metastasis | Distant Metastasis | Stage |
| --- | --- | --- | --- | --- | --- | --- | --- |
| 1 | Male | 71 | PASC | 5*4.1 | No | Yes | T3N0M1 |
| 2 | Male | 62 | PAAD | 2.6*1.8 | No | No | T2N0M0 |
| 3 | Female | 73 | PAAD | 2.6*2.1 | No | No | T2N0M0 |
| 4 | Female | 67 | PMN | 2.9*2.6 | Yes | No | T2N1M0 |
| 5 | Male | 66 | PAAD | 1.8*1.7 | No | No | T1N0M0 |
| 6 | Female | 64 | PAAD | 2.0*1.6 | No | No | T2N0M0 |

PASC, pancreatic adenosquamous carcinoma; PAAD, pancreatic adenocarcinoma; PMN, pancreatic mucinous neoplasm; T, tumor; N, node; M, metastasis
